# Aquagenic syringeal acrokeratoderma in an adolescent female with COVID-19

**DOI:** 10.1590/0037-8682-0152-2021

**Published:** 2021-04-12

**Authors:** Handan Alay, Handan Bilen

**Affiliations:** 1 Ataturk University, Faculty of Medicine, Department of Infectious Diseases and Clinical Microbiology, Erzurum, Turkey.; 2 Ataturk University, Faculty of Medicine, Department of Dermatology, Erzurum, Turkey.

**FIGURE 1:**
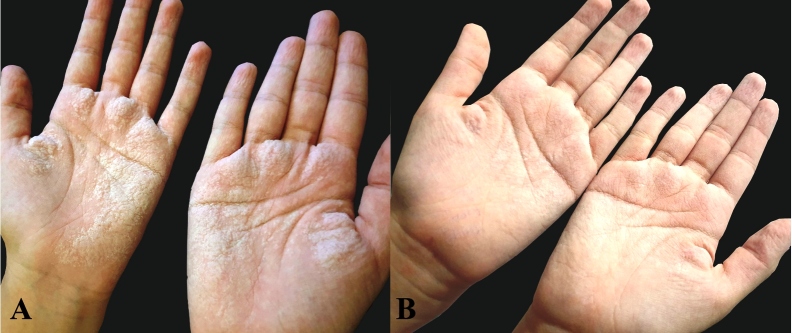
**(A):** Hypopigmentation in the palmar regions after contact with water accompanied by thickening of the skin, and the appearance of pronounced palmar creases and symmetrical, white-shiny papules (diameter, 1 mm). (**B).** The lesions appear to have regressed two months later.

**FIGURE 2:**
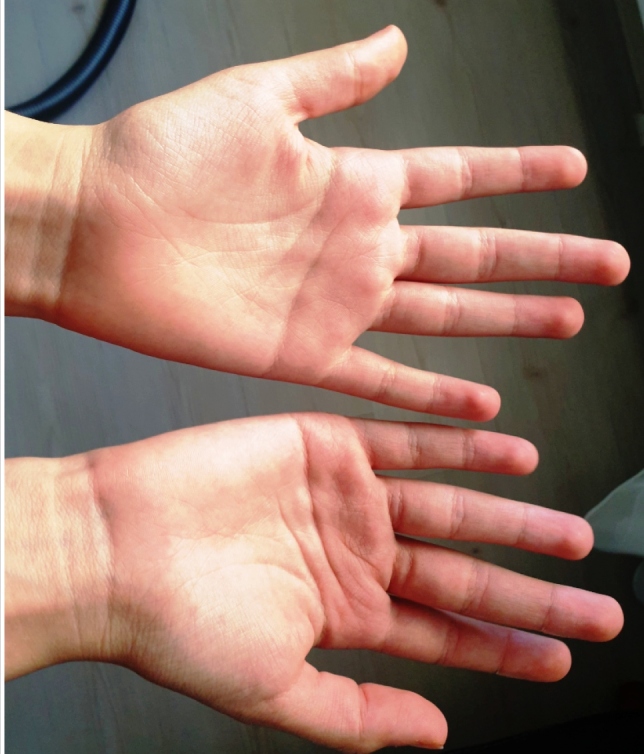
The hands appeared completely healed and healthy after following the recommendations for 3 months.

A 14-year-old female patient reported stinging and pain following contact with water and presented with raised white eruptions 10 days after being diagnosed with COVID-19. The patient reported that the eruptions appeared within 5 min after bathing and disappeared in 2-3 h. She had no history of plantar involvement, skin diseases, atopy, drug use, compression or trauma. Dermatological examination revealed that the following changes occurred upon contact with water: hypopigmentation in both palmar regions, thickening of the skin, and the appearance of pronounced palmar creases and symmetrical, white-shiny papules (diameter, 1 mm) (Figure 1A). Physical examination and laboratory test findings were unremarkable. Suspecting that the lesions may have been caused by frequent handwashing during the COVID-19 pandemic, the patient was advised to avoid using disinfectants unless absolutely necessary and to use soap and water for washing. Two months after following this advice, the lesions appeared to have regressed (Figure 1B), and by the third month, the lesions did not appear upon contact with water (Figure 2).

Aquagenic syringeal acrokeratoderma (ASA) is a rare entity generally affecting the palms of the hands and soles of the feet. ASA may be associated with cystic fibrosis, asthma, allergic rhinitis, a COX-2 inhibitor, and spironolactone use^1^ ^,^ ^2^. ASA has been reported following frequent exposure to water during the COVID-19 pandemic^3^.

Although rare, the possibility ASA development following excessive exposure to disinfectants that adversely affect the skin barrier should not be forgotten during this pandemic.
